# Are migratory waterfowl vectors of seagrass pathogens?

**DOI:** 10.1002/ece3.6039

**Published:** 2020-02-04

**Authors:** Damian Michael Menning, David Hume Ward, Sandy Wyllie‐Echeverria, George Kevin Sage, Megan Cathleen Gravley, Hunter Alexander Gravley, Sandra Looman Talbot

**Affiliations:** ^1^ Alaska Science Center US Geological Survey Anchorage AK USA; ^2^ Friday Harbor Laboratories College of the Environment University of Washington Friday Harbor WA USA; ^3^ US Fish and Wildlife Service Anchorage AK USA

**Keywords:** eDNA, eelgrass, pathogen, waterfowl

## Abstract

Migratory waterfowl vector plant seeds and other tissues, but little attention has focused on the potential of avian vectoring of plant pathogens. Extensive meadows of eelgrass (*Zostera marina*) in southwest Alaska support hundreds of thousands of waterfowl during fall migration and may be susceptible to plant pathogens. We recovered DNA of organisms pathogenic to eelgrass from environmental samples and in the cloacal contents of eight of nine waterfowl species that annually migrate along the Pacific coast of North America and Asia. Coupled with a signal of asymmetrical gene flow of eelgrass running counter to that expected from oceanic and coastal currents between Large Marine Ecosystems, this evidence suggests waterfowl are vectors of eelgrass pathogens.

## Graphical abstract

Migratory waterfowl vector plant seeds and other tissues, but little attention has focused on the potential of avian vectoring of species pathogenic on plants. When coupled with a signal of asymmetrical gene flow of eelgrass running counter to that expected from oceanic and coastal currents between Large Marine Ecosystems, our data support a model implicating waterfowl as vectors of plant pathogens. If waterfowl are vectoring eelgrass pathogens during their annual migratory cycles care must be taken when selecting seagrass restoration sites.

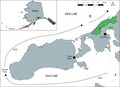

## 1 INTRODUCTION

**Figure 1:**
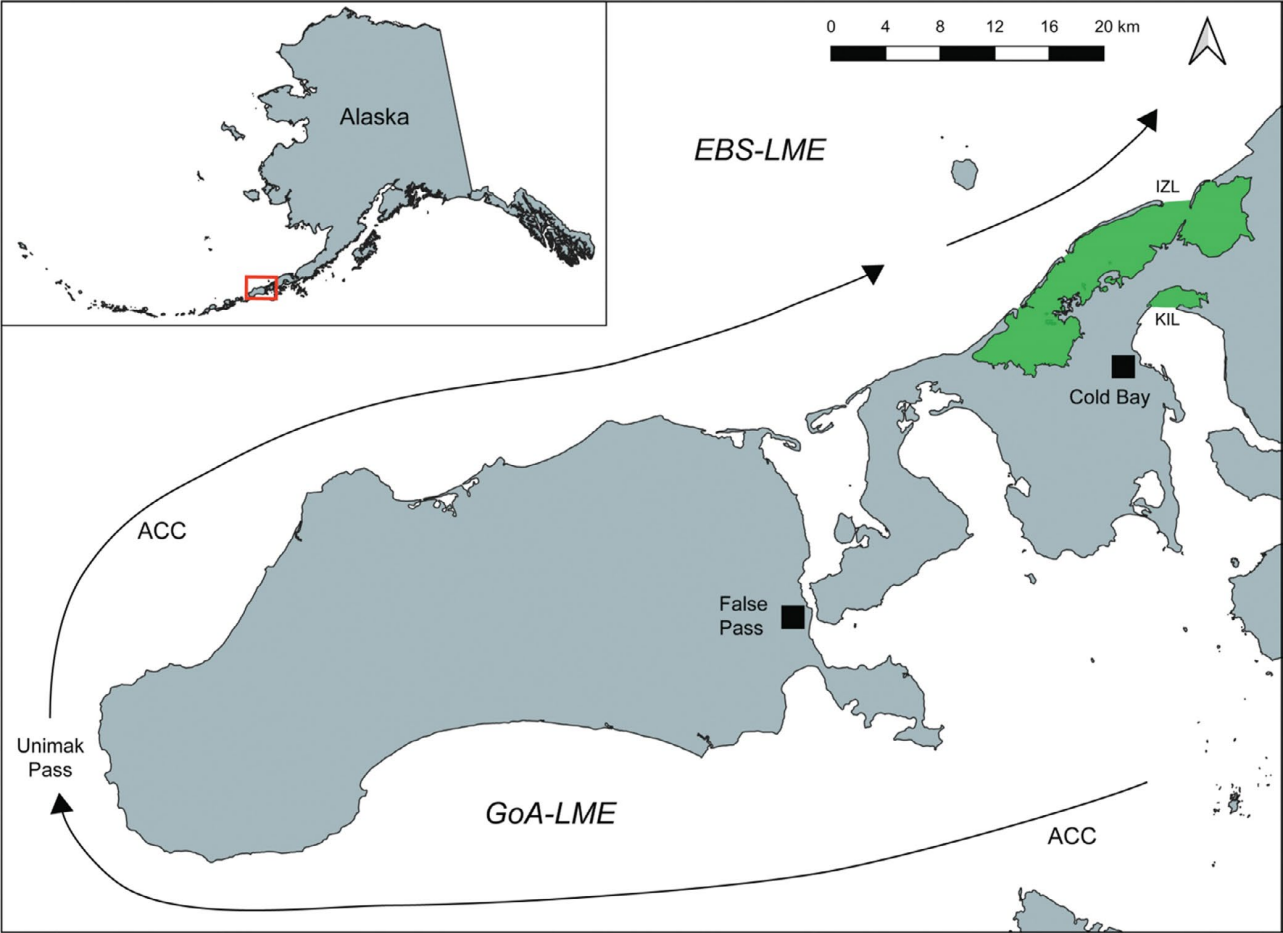
Environmental DNA sampling locations. Environmental DNA sample locations (in green) and prevailing direction of the Alaska Coastal Current (ACC) in the Gulf of Alaska Large Marine Ecosystem (GoA‐LME) and Eastern Bering Sea Large Marine Ecosystem (EBS‐LME). IZL, Izembek Lagoon; KIL, Kinzarof Lagoon. Green indicates extent of eelgrass meadows in IZL and KIL

Seagrasses comprise the most widespread coastal ecosystems in the world (Green & Short, 2003). At northern high latitudes, eelgrass (*Zostera marina*) is the predominant seagrass, providing critical ecosystem services and food for migrating waterfowl and other wild species (Arasaki, 1950; McRoy, 1968; Reed, Stehn, & Ward, 1989; Ward, Markon, & Douglas, 1997; Ward et al., 2005; Wyllie‐Echeverria, 1999). The extensive beds of eelgrass in southwest Alaska, particularly the Alaska Peninsula (Figure 1), support hundreds of thousands of waterfowl during fall migration (King & Dau, 1981; King & Derksen, 1986; Wilson, 2019) including the entire Pacific Flyway population of Black Brant (*Branta bernicla nigricans*). Black brant feed almost exclusively on eelgrass leaves and seeds prior to long‐distance migration to more southerly seagrass embayments along the Pacific coast (Derksen, Bollinger, Ward, Sedinger, & Miyabayashi, 1996; Lewis, Ward, Sedinger, Reed, & Brant, 2013).

Migratory birds play an important role as “global dispersal vectors” of organisms (Derksen et al., 1996; Viana, Santamaría, & Figuerola, 2016), including pathogens and parasites, and much research has been directed toward the vectoring of zoonoses and wildlife disease by migratory bird species nesting in northern high latitudes (Van Hemert, Pearce, & Handel, 2014; Viana et al., 2016). Further, in some cases, gene flow among eelgrass forming meadows in embayments along the Alaska Peninsula is inconsistent with dispersal via oceanic currents and is consistent with vectoring by waterbirds (Talbot et al., 2016). Thus, waterfowl likely play a major role in the short‐distance dispersal of eelgrass via endochory (Kleyheeg et al., 2019; Sanchez, Green, & Castellanos, 2006; Wyllie‐Echeverria, 1999). While avian species are known to vector plant seeds and other tissues (Leeuwen, Velde, Lith, & Klaassen, 2012), little attention has focused on the potential of avian vectoring of species pathogenic on plants.

In the 1930s, eelgrass endured an almost complete die‐off of the species' Atlantic distribution due presumably to a virulent pathogenic strain of protist, *Labyrinthula zosterae.* This outbreak resulted in declines in eelgrass‐dependent fish and waterfowl species (Short, Muehlstein, & Porter, 1987), including a collapse of the Atlantic Flyway population of Atlantic Brant, *B. b. hrota* (Cottam, Lynch, & Nelson, 1944; Kirby & Obrecht, 1982); however, *L. zosterae* is not the only pathogenic organism known to impact eelgrass health. In 2016, Govers et al. (2016) reported the presence of two other closely related fungi‐like oomycete species associated with eelgrass in the Atlantic: *Phytophthora gemini* and a previously undescribed species, *Halophytophthora* sp*.* Zostera. Both oomycete species are potent pathogens closely related to the potato blight (*P. infestans*), and both may strongly reduce sexual reproduction in eelgrass by reducing seed germination (Govers et al., 2016). Diseases specific to eelgrass continue to play an important role in regulating eelgrass populations in Europe and the Atlantic coast of North America (Bishop, Martin, & Ross, 2017; Govers et al., 2016; Muehlstein, 1989; de los Santos et al., 2019; Short et al., 1987) and may play a future role along the Pacific coast (Martin et al., 2016).

Current research indicates eelgrass pathogens may be transmitted via direct contact with infected tissue and/or debris (Martin et al., 2016; Muehlstein, 1992) or via waterborne transmission of other infected substrates (Martin et al., 2016). It is unclear how these eelgrass pathogens are vectored in the north Pacific, although certainly oceanographic and coastal currents play a role in eelgrass dispersal (Kendrick et al., 2012), and so presumably in the dispersal of associated pathogens. Nevertheless, a signal of counter‐current gene flow between certain eelgrass meadows along the Alaska Peninsula suggests oceanic and coastal currents alone are not the only dispersal mechanism for eelgrass populations in the region (Talbot et al., 2016). Further, there is an unexpectedly close genetic relationship between eelgrass meadows in Kinzarof and Izembek lagoons (Talbot et al., 2016), two lagoons separated by only 5 km of land, but at the least 510 km of coastline via the Alaska Coastal Current (ACC) that flows northward from the Gulf of Alaska Large Marine Ecosystem (GoA‐LME) into the Eastern Bering Sea Large Marine Ecosystem (EBS‐LME) at Unimak Pass (Figure 1). This finding suggests an additional dispersal mechanism—in particular, waterfowl (Talbot et al., 2016)—for eelgrass, and thus for pathogens on eelgrass.

**Figure 2:**
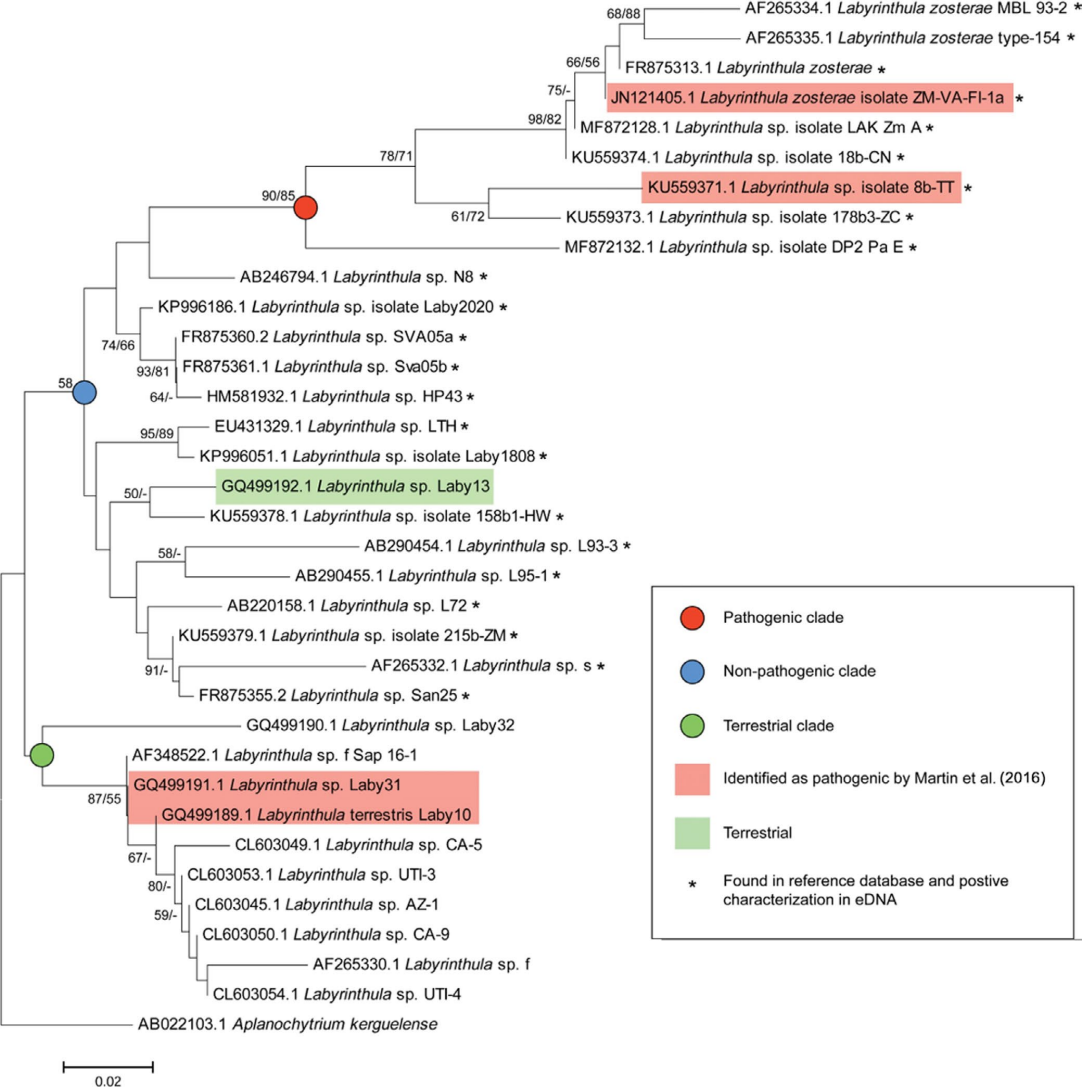
*Labyrinthula* spp. phylogeny*. Labyrinthula* spp. phylogeny, based on partial 18S rDNA sequences, reconstructed from Martin et al. (2016) and replicating their analyses. Maximum‐likelihood (1,000×) and neighbor joining (500×) bootstrap values are shown in the nodes, respectively. Red circles in the node identify the pathogenic clade, and OTUs, designated by GenBank Accession numbers corresponding to nucleotide sequence; highlights in red indicate strains known to be pathogenic based on Martin et al. (2016). OTUs designated by asterisks represent sequences recovered from environmental samples collected from Grant Point, Izembek Lagoon, AK

To investigate potential links between migratory bird species, eelgrass communities, and vectors of eelgrass pathogens along the Alaska Peninsula, we leveraged environmental DNA metabarcoding to (a) detect the presence of DNA of both classes of pathogens in environmental samples (sediment, water column, plant), (b) test for seasonal differences in their presence, and (c) determine whether DNA from eelgrass pathogens (Figure 2) occurred in the cloaca of waterfowl species that forage on eelgrass. The presence of eelgrass pathogens in cloacal contents of waterfowl would suggest the potential for vectoring of the pathogens among embayments via endochory—that is, when plants are eaten and dispersed by animals. To test this hypothesis, we analyzed gene flow (Beerli & Felsenstein, 1999, 2001) based on microsatellite loci genotyped from eelgrass collected in Izembek and Kinzarof lagoons. A finding of eelgrass pathogens in Izembek Lagoon and in cloacal contents of waterfowl species, coupled with a signal of asymmetrical gene flow in eelgrass running counter to that expected—northward from Kinzarof Lagoon to Izembek Lagoon—would suggest a model implicating waterfowl as vectors of eelgrass pathogens.

## 2 MATERIALS AND METHODS

### 2.1 Sample collection

Environmental samples were collected from the water column, sediment, and eelgrass leaves at Grant Point, Izembek Lagoon (55°16′12.40″N, 162°53′50.58″W), and from the cloaca of waterfowl species that had recently foraged in eelgrass meadows in Izembek Lagoon. Water samples were collected in 500 ml volumes and filtered through 0.22‐micron filters (GTTP 04700, Millipore), which were then stored in 5 ml of Longmire Buffer (Longmire, Maltbie, & Baker, 1997) held in 15‐ml falcon tubes (Menning, Simmons, & Talbot, 2018). Sediment samples were collected in 1 ml volumes and stored in 15‐ml tubes containing 5 ml of LMB. Approximately three inches of plant tissue (leaf) were collected and stored in 15‐ml tubes containing 5 ml of LMB. Cloacal samples, which at times included seeds, were collected opportunistically from sport hunters that shot birds in the Cold Bay, AK, area during fall 2016 (Black Brant; Northern Pintail, *Anas acuta*) and fall 2018 (Black Brant; Cackling Goose, *B. hutchinsii*; Emperor Goose, *Anser canagicus*; Northern Pintail; American Wigeon, *A. americana*; Eurasian Wigeon, *Mareca penelope*; Green‐winged Teal, *A. crecca*; Mallard, *A. platyrhynchos*; and Greater Scaup, *Aythya marila*) using sterile foam‐tipped applicators and stored in 15‐ml tubes containing 5 ml of LMB. Five replicates of each sample type were collected at each location unless otherwise noted.

### 2.2 DNA extraction

Stored environmental samples were vortexed and eDNA extracted using a 400 µl subsample of the LMB‐preserved sample using a Qiagen DNeasy Blood and Tissue kit (Qiagen), following manufacturers suggested protocols, with the exception that volumes were doubled. To avoid contamination, all extractions were conducted in a laboratory in which polymerase chain reactions (PCRs) have never been conducted and which is separated physically from laboratories where PCRs are conducted. Additionally, prior to this study, no studies involving seagrass pathogens were ever conducted in this laboratory, or any other laboratories located in the same facility.

### 2.3 Primer and reference database design

Primers were designed using Python (Van Rossum, 1995) and Biopython (Cock et al., 2009) scripts that are part of the U.S. Geological Survey Alaska Science Center Bioinformatics pipeline (Menning & Talbot, 2018). Briefly, all available *Labyrinthula* sp., *Halophytophthora* sp., and *Phytophthora* sp. sequences were downloaded from NCBI GenBank on 11 October 2018 into locus‐specific FASTA files (5.8S and 18S). Each FASTA file was aligned using MEGA6 (Tamura, Stecher, Peterson, Filipski, & Kumar, 2013). Aligned FASTA files were used to locate potential primer sites (conserved regions greater than 17 base pairs). Once candidate primers were identified, they were checked against all locus‐specific FASTA sequences on NCBI. The resultant FASTA files were screened to verify that a single taxon would be identified for each unique sequence and that no potential sequences had more than one unique generic/specific epithet descriptor (primer sequences are listed in Table 1). Locus‐specific primers were appended with Illumina Nextera XT (Illumina Inc.) adapters without indices and synthesized by Eurofins Genomics (http://www.eurofinsgenomics.com).

**Table 1:** Taxon‐specific pathogen primers designed for this study

Target species	Locus	Forward primer sequence (5′‐3′)	Reverse primer sequence (5′‐3′)
*Labyrinthula* sp.	5.8S	CAATGAATATCTTGGTTTCCG	GAGTGCTCGTTTGTGGACG
*Labyrinthula* sp.	18S	ACCACATCCAAGGAAGGC	AATATACGCTACTGGAGC
*Halo/Phytophthora* spp.	ITS	AACTTTCCACGTGAACCG	TAAAAGCAGAGACTTTCG
*Phytophthora* sp*.*	COI	TCDTCDHTATTAGGTGC	GTRTTWAARTTTCTATC

Note

Loci 5.8S and 18S are nuclear small and large ribosomal RNA subunit genes; ITS refers to the internal transcribed spacer DNA situated between the large and small‐subunit RNA genes. COI refers to the mitochondrial cytochrome oxidase 1 gene.

The reference database was developed by downloading all sequences that matched the search criteria “target locus” and one of the following (“eukaryotes”[porgn:__txid2759], “oomycetes”[porgn:__txid4762], “slime nets”[porgn:__txid35131]), and included all sequences in Martin et al. (2016), which provides sequence data associated with virulence in *Labyrinthula*. Following protocols reported in Menning et al. (2018), each downloaded sequence was cropped to the 5′ end of each locus‐specific primer, aligned using MEGA6, and checked to verify correct alignment with the target reference sequences, were not duplicated (one of each identical pair was culled), and did not coamplify nontarget species. Although our main focus was to target species known to be pathogenic, we also included in the final reference database sequence data from *Labyrinthula* strains thought to be nonpathogenic (Table 2), to verify the results published by Martin et al. (2016).

**Table 2:** GenBank accession numbers used in custom reference database

KU559371.1
MF872132.1
MF872128.1
KU559373.1
KU559375.1
AF265334.1
AF265335.1
KU559377.1
KU559410.1
KU559419.1
KU559420.1
KU559388.1
JN121409.1
MF326859.1
KT986007.1
KX172080.1
NR_147866.1
KX172082.1
MG865498.1

### 2.4 DNA library preparation and sequencing

Environmental DNA libraries were prepared using a two‐step PCR protocol and sequenced using an Illumina MiSeq. PCR reaction master mix per sample consisted of 0.2 µl molecular grade water (Thermo Fisher Scientific), 5.0 µl Phusion High‐Fidelity PCR Master Mix with HF Buffer (New England BioLabs), 0.8 µl Molecular Grade BSA (20 mg/µl, New England BioLabs), 1.0 µl of each forward and reverse primer, and 2.0 µl eDNA. A PCR was conducted using each eDNA sample with each locus‐specific primer separately under the following conditions: 98°C for 30 s, followed by 40 cycles of 98°C for 10 s, 52°C for 30 s, and 72°C for 40 s, with a final elongation step at 72°C for 5 min. Following the first PCR, excess primers and dNTPs were removed using ExoSap (Affymetrix), and all loci were quantified by fluorometry using a Quant‐IT Broad Range kit (Thermo Fisher Scientific), normalized to equal concentrations, and pooled by sample. To produce barcoded amplicons, a second PCR was performed on each locus‐pooled sample consisting of 2.0 µl molecular grade water (Thermo Fisher Scientific), 5.0 µl Phusion High‐Fidelity PCR Master Mix with HF Buffer (New England BioLabs), 1.0 µl of each I5 and I7 Nextera XT index primer, and 1.0 µl pooled PCR product under the following conditions: 98°C for 30 s, followed by 20 cycles of 98°C for 10 s, 60°C for 30 s, and 72°C for 40 s, with a final elongation step at 72°C for 5 min. All second‐step PCR products were quantified by fluorometry using a High Sensitivity Quant‐IT kit (Thermo Fisher Scientific), normalized to equal concentrations, and pooled. Quantified pooled PCR products were electrophoresed on a 1% polyacrylamide gel to estimate PCR fragment sizes and gel‐purified using Qiagen Gel purification kit (Qiagen). Gel‐purified PCR products were quantified by fluorometry using a Quant‐IT High Sensitivity kit (Thermo Fisher Scientific), diluted to 2 nM concentrations following Illumina guidelines (Illumina Document # 15039740 v01), requantified by fluorometry using a Quant‐IT High Sensitivity kit, and further diluted to 20 pM following the Illumina NextSeq Protocol A (Illumina Document #15048776 v02) for library dilution. All remaining steps for library preparation followed Illumina MiSeq protocols (Illumina Part #15034097 Rev. B). The eDNA library and PhiX were subsequently diluted to 15 pM. Sequencing was performed using an Illumina MiSeq 300 cycle v2 reagent kit (2 × 151 paired‐end cycle runs, Illumina Part #MS‐102‐2002) on an Illumina MiSeq with a 30% PhiX spike.

### 2.5 Bioinformatic analyses

All demultiplexed data were retrieved from the Illumina MiSeq and analyzed in the same manner as Menning et al. (2018) with the exception that the default BLAST+ parameters reward/penalty were changed to 1/−3, respectively, and the gapopen/gapextend parameters were set at 1/1 to ensure at least a 99% match to the reference database. Quality filtering to remove sequencing errors was conducted by including only match count information that exceeded 0.01% of the total number of reads passing filter, per sample, in the MiSeq run (Bokulich et al., 2013). Bioinformatic analyses were conducted using the USGS Yeti Supercomputer (USGS Advanced Research Computing, 2000).

### 2.6 Assignment of pathogenicity of *Labyrinthula*

To assign any reads to the pathogenic versus nonpathogenic *Labyrinthula* clades, we reanalyzed sequence reads that were included in the phylogenetic tree comprising Figure 3 in Martin et al. (2016), which were identified to pathogenicity (see Figure 2). This facilitated our ability to estimate whether any *Labyrinthula* sequences recovered in our eDNA analysis could be assigned to virulent versus nonvirulent forms.

**Figure 3:**
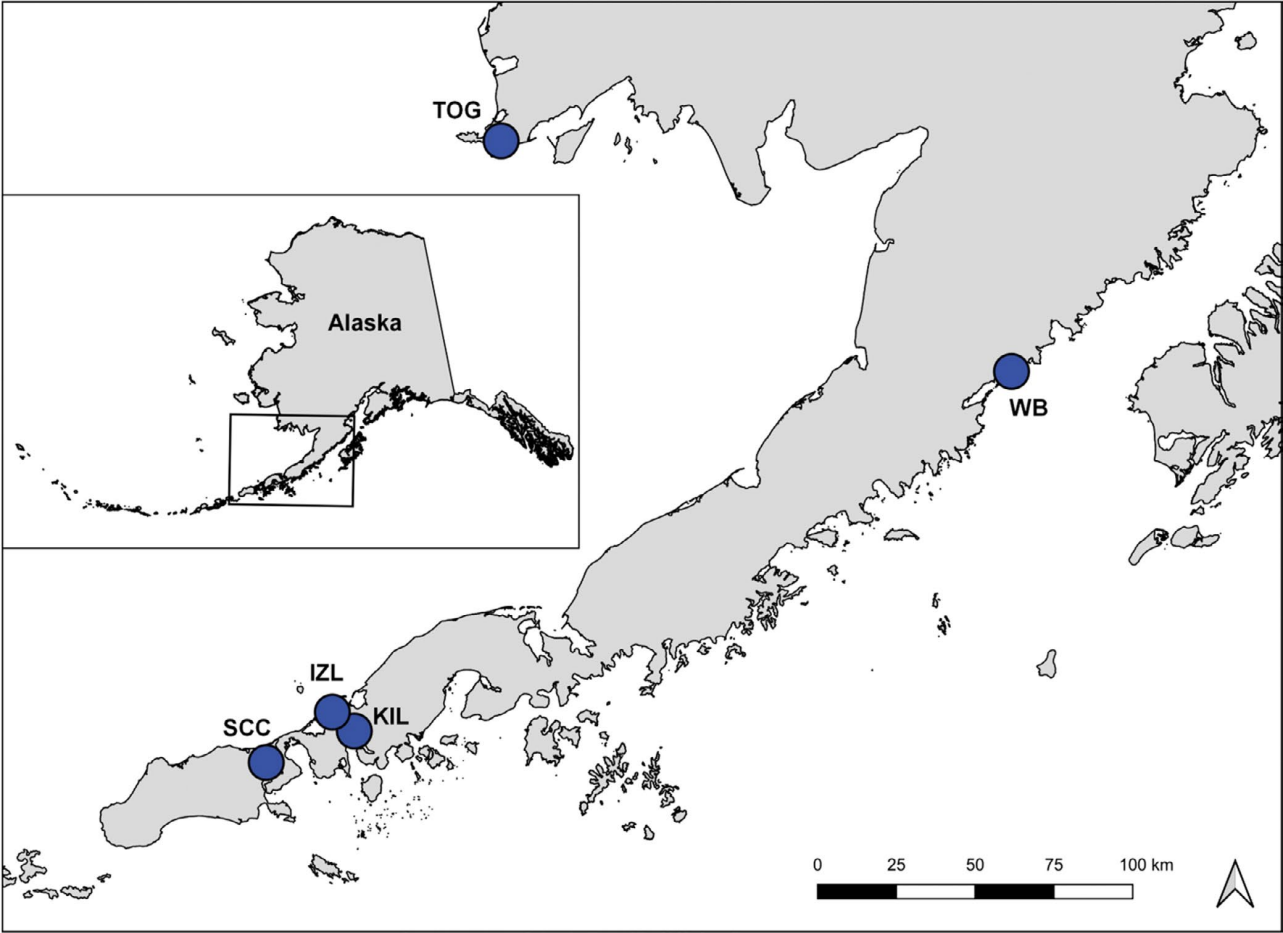
Eelgrass collection sites. Collection sites for eelgrass (*Zostera marina*) samples used in microsatellite data collection for gene flow polarity analysis

### 2.7 Gene flow rates and polarity for eelgrass (*Zostera marina*)

We estimated the magnitude and polarity of gene flow among populations within the two regions using the maximum‐likelihood approach implemented in MIGRATE 3.6.8 (Beerli & Felsenstein, 1999, 2001). MIGRATE uses a coalescence approach to estimate mutation‐scaled immigration rates, *M*, among populations (*M* = *m*/*mμ*, where *m* = immigration rate per generation and *μ* = mutation rate per generation), assuming a constant per‐locus mutation rate (*μ*). This approach is judged to estimate gene flow more accurately than other F_ST_ methods, especially when multiple loci are employed (Beerli & Felsenstein, 1999). The program assumes discrete populations and generations, mutation‐drift equilibrium, no selective effects, and the SMM for microsatellite markers. Given the net gene flow between GoA‐LME is northward into the EBS‐LME, we expect gene flow estimates should be asymmetrical from Kinzarof to Izembek lagoon.

**Figure 4:**
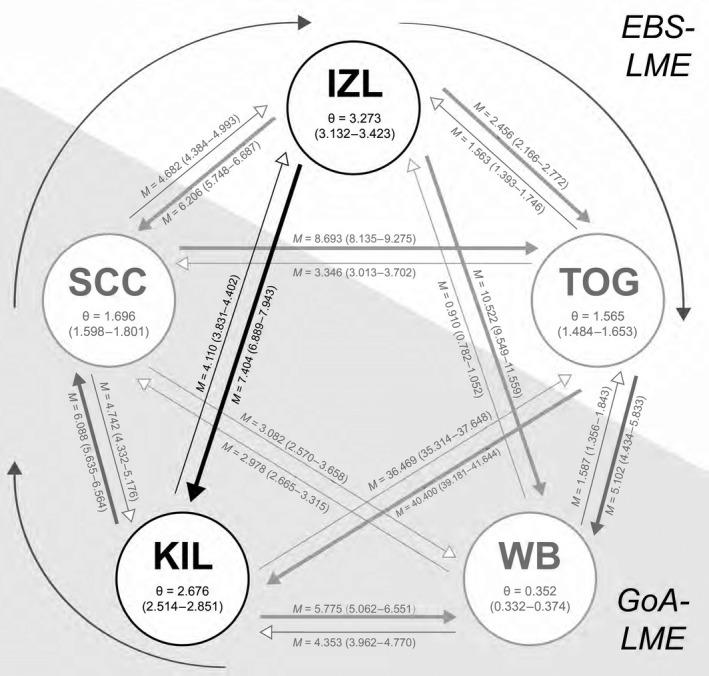
Schematic of results of gene flow analyses. Schematic of results of gene flow analyses, based on a full model migration matrix (all parameters allowed to vary independently) among five populations of eelgrass (*Zostera marina*), two (KIL, WB) in the Gulf of Alaska Large Marine Ecosystem (GoA‐LME, gray background), and 3 (SCC, IZL, TOG) in the Eastern Bering Sea LME (EBS‐LME; white background). We calculated gene flow polarity and rates based on 10 microsatellite loci (Talbot et al., 2016). *M* = mutation‐scaled immigration rates; Θ = population size (4*N*_e_ *μ*). Ninety‐five percent confidence intervals for parameter values are in parentheses. Arrow thickness is proportionate to estimated levels of gene flow (thicker arrows indicate higher relative gene flow). IZL, Izembek Lagoon; KIL, Kinzarof Lagoon; SCC, Saint Catherine Cove; TOG, Togiak Bay; WB, Wide Bay. Curvilinear arrows around the perimeter of the figure indicate net direction of oceanic flow within and between the LMEs. Data used for this analysis can be found at http://dx.doi.org/10.5066/F7GQ6VTK

We performed MIGRATE analyses using data from 10 microsatellite loci, gathered as part of prior research (Talbot et al., 2016; see http://dx.doi.org/10.5066/F7GQ6VTK) from two meadows located in the GoA‐LME and three meadows located in the EBS‐LME. Meadows in the GoA‐LME included Kinzarof Lagoon (KIL), located on the southern tip of the Alaska Peninsula (see Figure 1), and Wide Bay (WB), located along the middle southern coast of the Alaska Peninsula. Meadows in the EBS‐LME included Izembek Lagoon (IZL), located on the northern tip of the Alaska Peninsula (see Figure 1), Saint Catherine Cove (SCC), slightly west of IZL, and Togiak Bay (TOG), located along the southwestern coast of Alaska (see Figure 3 for all sampling sites). Full models, Θ = 4*N*_e_ *μ* (the composite measure of effective population size and mutation rate, where *N*_e_ = effective population size), and all pairwise migration parameters were estimated individually from the data. Significant asymmetry in gene flow was assessed by comparing 95% confidence intervals, where nonoverlapping confidence intervals in values of *M* indicated significant asymmetry in immigration rates between population pairs. MIGRATE was performed using maximum‐likelihood search parameters (10 short chains using 1,000 trees of 25,000 sampled followed by five long chains using 10,000 trees out of 250,000 sampled and five adaptively heated chains (start temperatures: 1, 1.5, 3, 6 and 12; swapping interval = 1). To ensure convergence of parameter estimates, full models were run ten times. We reported the results of one representational MIGRATE analysis in the text (also see Figure 4) and reported the results of MIGRATE analyses below for pairwise estimates between Kinzarof Lagoon and Izembek lagoons. MIGRATE analyses were conducted using USGS Yeti Supercomputer (USGS Advanced Research Computing, 2000).

## 3 RESULTS

### 3.1 Estimates of gene flow rates and polarity for eelgrass (*Zostera marina*)

**Figure 5:**
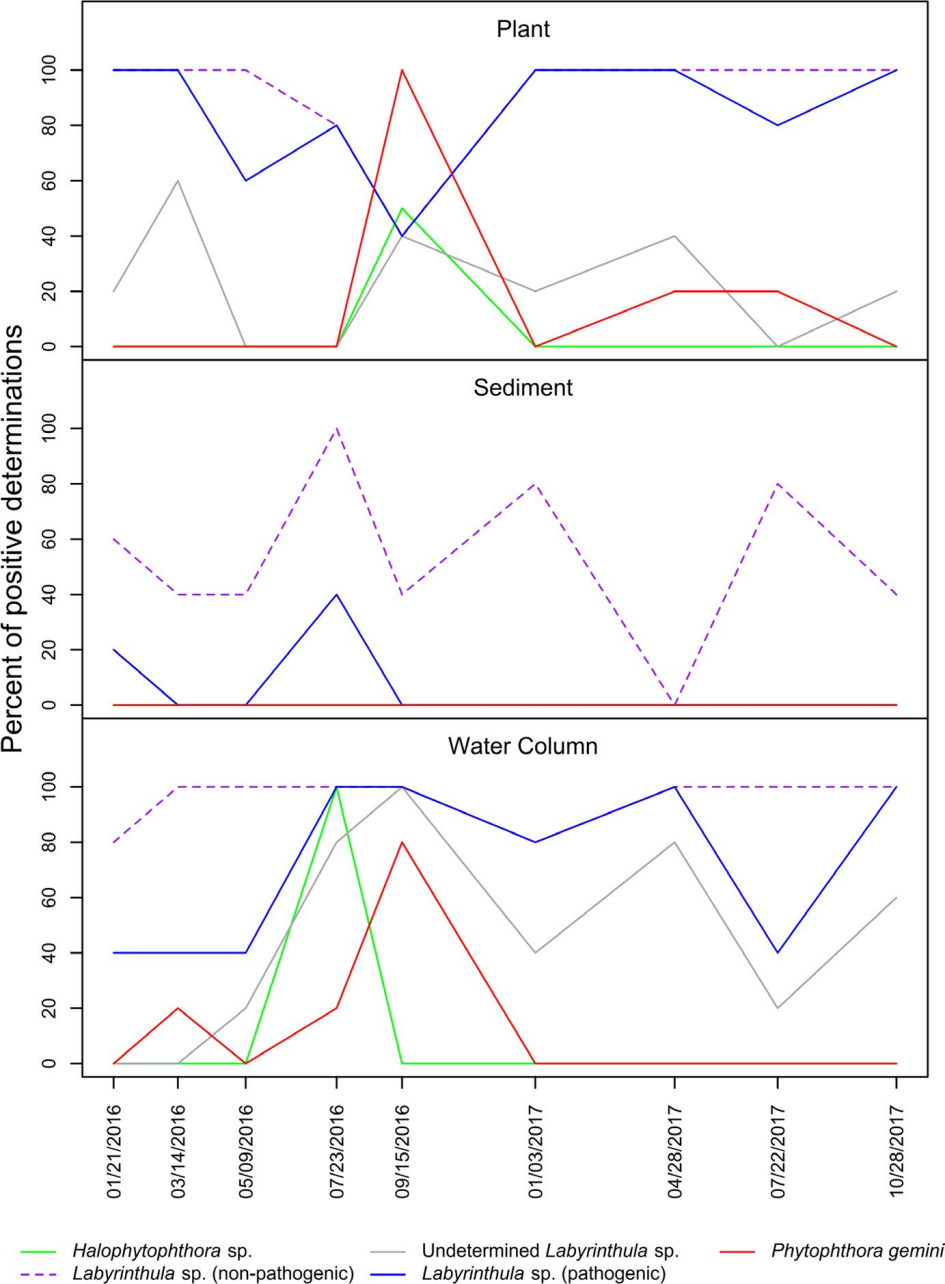
*Zostera marina* pathogens found in environmental DNA samples. Percent of positive determinations of DNA from organisms with strains known to be pathogenic on eelgrass, found in environmental samples (plant leaf, sediment, and water column) collected from Grant Point, Izembek Lagoon, AK, during 2016 and 2017. Dates of collection are provided on the *x*‐axis (m/d/y), and percent of positive determinations among five samples per location are given on the *y*‐axis for each environmental sample type

**Figure 6:**
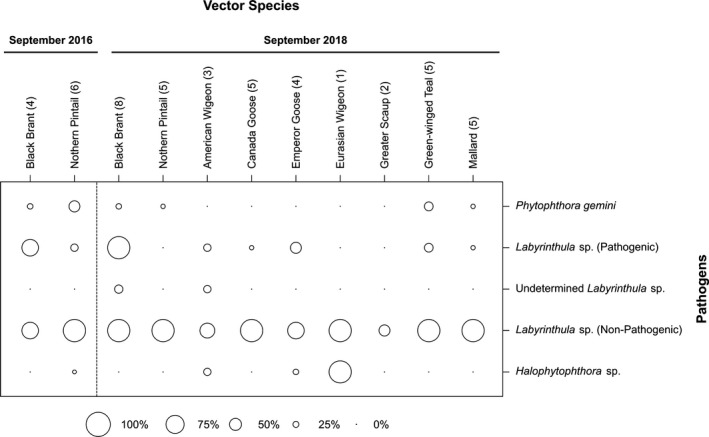
*Zostera marina* pathogens found in cloacal contents of migratory waterfowl. Percent of positive determinations of DNA from organisms with strains known to be pathogenic on eelgrass, found in cloacal contents of two species of waterfowl harvested by hunters in Cold Bay, Alaska, during September 2016, and nine species of waterfowl harvested during September 2018

Estimates of gene flow rates and polarity (Figure 4) suggested significant asymmetrical gene flow between eelgrass meadows in Izembek and Kinzarof lagoons; maximum‐likelihood estimates of mutation‐scaled immigration rates, *M*, based on 10 Markov Chain Monte Carlo simulations, imply that the direction of gene flow was predominantly from Izembek Lagoon southward to Kinzarof Lagoon (*θ* = 3.273, 95% CI = 3.132–3.423, *M*_IZL→KIL_ = 7.404, 95% CI = 6.889–7.943; *M*_KIL→IZL_ = 4.110, 95% CI = 3.831–4.402; Table 3). Results of one representative gene flow analysis (run 1, above) for all five populations are shown in Figure 4. Extensive annual and seasonal variation of presence was uncovered for the oomycete species in Izembek Lagoon. *Halophytophthora* sp. was only detected in Izembek Lagoon during the summer of 2016 (Figure 5), and in cloacal contents sampled from Northern Pintail in 2016, and American Wigeon, Eurasian Wigeon, and Emperor Goose during fall (September) 2018 (Figure 6). Reads from *P. gemini* were detected in samples collected in spring and summer from the water column or eelgrass, and from Black Brant and Northern Pintail sampled in fall 2016 and 2018, as well as in Green‐winged Teal and Mallards in fall 2018 (Figure 6). In contrast, both nonpathogenic and pathogenic *Labyrinthula* strains were found in environmental samples in each sampling period (January, March, July, and September 2016, and January, April, July, October 2017, and August 2018) with the percentage of positive determinations increasing in the sediment during summer months (Figure 5). Reads from nonpathogenic *Labyrinthula* strains occurred in the cloacal contents of every waterfowl species assayed, and pathogenic strains were present in all species except Northern Pintail, Eurasian Wigeon, and Greater Scaup (Figure 6).

**Table 3:** Maximum‐likelihood estimates of *M* (*M* = *m*/*mμ*, where *m* = immigration rate per generation and *μ* = mutation rate per generation), based on Markov Chain Monte Carlo simulations for all 10 simulations

Run	IZL			KIL			IZL–KIL			KIL–IZL		
	0.05	Theta	0.95	0.05	Theta	0.95	0.05	M	0.95	0.05	M	0.95
1	3.132	3.273	3.423	2.514	2.676	2.851	6.889031	7.4035	7.94322	3.830543	4.1095	4.401949
2	3.436323	3.59759	3.769031	2.460823	2.61523	2.782831	8.183719	8.7048	9.247373	4.480709	4.7891	5.11151
3	3.289603	3.44211	3.604222	3.132539	3.34499	3.577218	6.423371	6.8866	7.371539	3.21865	3.4707	3.735489
4	3.036516	3.17315	3.318168	1.818832	1.94323	2.079327	11.27847	12.0411	12.83582	3.953188	4.2335	4.526693
5	3.535774	3.69996	3.874462	3.340085	3.55441	3.787434	8.458144	8.9813	9.525672	5.616566	5.9483	6.293007
6	2.875197	3.01288	3.159473	1.922995	2.04333	2.173953	7.087407	7.6302	8.199998	4.236965	4.5748	4.93008
7	2.884814	3.01344	3.149888	3.259797	3.50687	3.779519	6.126148	6.6386	7.178617	4.475726	4.8001	5.139941
8	4.512579	4.72305	4.946801	1.654019	1.76163	1.878795	9.216334	9.8851	10.5848	3.469865	3.7232	3.988601
9	3.443705	3.59502	3.755373	2.88341	3.08186	3.298995	6.347591	6.8273	7.330762	2.481547	2.6924	2.914834
10	3.46892	3.62539	3.791399	2.876841	3.05583	3.250711	4.965937	5.3787	5.813804	2.321261	2.545	2.777587

### 3.2 Environmental DNA

This study reports the first instance in Alaska waters of pathogenic strains of *Labyrinthula* known to cause declines in seagrass meadows along the Atlantic coast of North America and Europe (Groner et al., 2014, 2016) and two other seagrass pathogens, *Phytophthora gemini* and *Halophytophthora* sp*.* Zostera. *Labyrinthula* strains were detected in cloacal contents of eight of nine waterfowl species that annually migrate along the Pacific coast of North America and Asia (Derksen et al., 1996; Koehler, Pearce, Flint, Franson, & Ip, 2008; Lane & Miyabayashi, 1997). Although plant seeds can remain viable after passing through the waterfowl digestive system (Leeuwen et al., 2012), finding sequence reads from pathogenic taxa in waterfowl cloacal samples does not demonstrate that the organisms themselves remain viable. Additional experiments are needed to demonstrate viability.

### 3.3 Bioinformatic analyses

All demultiplexed Illumina MiSeq data can be found at NCBI BioProject PRJNA548352, and sample information can be found in Menning et al. (2019). The percentage of samples that had a potentially pathogenic species or strain positively identified is shown for environmental samples collected from Izembek Lagoon (Figure 5) and waterfowl harvested by hunters in the Cold Bay, AK area (Figure 6). Percentage of positive identifications per sample are also shown for nonpathogenic *Labyrinthula* strains.

## 4 DISCUSSION

Izembek Lagoon is the first major intertidal eelgrass embayment waterfowl species encounter on the north side of the Alaska Peninsula during their southward migration from northerly breeding grounds. The lagoon contains the largest (16,000 ha) expanse of intertidal eelgrass on the Alaska Peninsula (Hogrefe, Ward, Donnelly, & Dau, 2014) and supports the greatest number of waterfowl in the region during fall migration (Wilson, 2019). During arrival, birds generally settle in Izembek Lagoon before moving on to Kinzarof Lagoon and other nearby eelgrass embayments on the peninsula (Boyd, Ward, Kraege, & Gerick, 2013). Therefore, the gene flow estimates are consistent with the hypothesis that eelgrass may be dispersed via endochory by waterfowl at levels sufficient to overcome the signal of gene flow facilitated by oceanic and coastal currents (Talbot et al., 2016), supporting a model for the transmission of associated pathogenic organisms via endochory. Thus, while gene flow and population dynamics for marine organisms, and presumably associated pathogens, are influenced by the distance between populations, currents, and oceanographic mixing patterns (Hedgecock, 1986), they may also be affected by movements of birds (Arasaki, 1950; Nacken & Reise, 2000; Sanchez et al., 2006; Sumoski & Orth, 2012).

The migratory waterfowl that stage on the lower Alaska Peninsula may consume large amounts of eelgrass (Lewis et al., 2013) and have large migratory ranges along the eastern and western sides of the Pacific (Derksen et al., 1996) that could have cascading ecological impacts on coastal marine communities. This intersection of multiple migratory ranges may facilitate the transmission of diseases outside of the range of any one particular migratory species. For example, Winker and Gibson (2010) found that migratory birds can move avian influenza from Asia to Alaska where they could infect other species and continue passing this infection across North America via other migratory pathways.

One of the earliest records of a marine conservation translocation involved eelgrass rhizomes translocated from the Pacific coast to the Atlantic of North America in 1943, following eelgrass decline in the Atlantic attributed to a pathogenic *Labyrinthula* infection (Cottam & Addy, 1947). In that instance, the successful transplants were destroyed by the disease the following year. While guidelines for translocation of eelgrass vary across regions, most recommend the selection of target restoration sites be located in areas where factors associated with eelgrass loss, including disease, are resolved and can be prevented (Evans & Leschen, 2009; Fonseca, Kenworthy, & Thayer, 1998). Ironically, the earliest guidelines for translocation of eelgrass beds following decline due to disease in the Atlantic recommended that transplants be “restricted to bays and estuaries…where waterfowl may be expected to feed” (Cottam & Addy, 1947; p. 397). However, if waterfowl are vectoring eelgrass pathogens during their annual migratory cycles, it is unclear how to prevent the introduction of disease pathogens into restored meadows.

## CONFLICT OF INTERESTS

Authors declare no competing interests.

## AUTHOR CONTRIBUTIONS

DMM, SLT, DHW, and SW‐E conceptualized the data. DMM curated the data. DMM and SLT performed formal analysis. SLT and DHW involved in funding acquisition, project administration, resources, and supervision. HAG, MCG, DMM, GKS, SLT, and DHW investigated the study. DMM, SLT, and DHW contributed to methodology. MCG, DMM, GKS, and SLT performed validation. HAG and MCG performed visualization. HAG, MCG, DMM, GKS, SLT, DHW, and SW‐E wrote—original draft.

## Data Availability

All demultiplexed Illumina MiSeq data can be found at NCBI BioProject PRJNA548352, and sample information will be made publicly available via data release upon publication acceptance. All other data are available in the main text.
